# Protein-segment universe exhibiting transitions at intermediate segment length in conformational subspaces

**DOI:** 10.1186/1472-6807-8-37

**Published:** 2008-08-13

**Authors:** Kazuyoshi Ikeda, Takatsugu Hirokawa, Junichi Higo, Kentaro Tomii

**Affiliations:** 1 Computational Biology Research Center (CBRC), National Institute of Advanced Industrial Science and Technology (AIST), 2-42 Aomi, Koto-ku, Tokyo 135-0064, Japan; 2 School of Life Science, Tokyo University of Pharmacy and Life Science, 1432-1 Horinouchi, Hachioji, Tokyo 192-0392, Japan; 3 Institute for Bioinformatics Research and Development (BIRD), Japan Science and Technology Agency, Chiyoda-ku, Tokyo 102-8666, Japan; 4 PharmaDesign, Inc., 2-19-8 Hacchobori, Chuo-ku, Tokyo 104-0032, Japan; 5 461 Koshland Hall, University of California, Berkeley, CA 94720-3102, USA

## Abstract

**Background:**

Many studies have examined rules governing two aspects of protein structures: short segments and proteins' structural domains. Nevertheless, the organization and nature of the conformational space of segments with intermediate length between short segments and domains remain unclear. Conformational spaces of intermediate length segments probably differ from those of short segments. We investigated the identification and characterization of the boundary(s) between peptide-like (short segment) and protein-like (long segment) distributions. We generated ensembles embedded in globular proteins comprising segments 10–50 residues long. We explored the relationships between the conformational distribution of segments and their lengths, and also protein structural classes using principal component analysis based on the intra-segment **C**_α_-**C**_α _atomic distances.

**Results:**

Our statistical analyses of segment conformations and length revealed critical dual transitions in their conformational distribution with segments derived from all four structural classes. Dual transitions were identified with the intermediate phase between the short segments and domains. Consequently, protein segment universes were categorized. i) Short segments (10–22 residues) showed a distribution with a high frequency of secondary structure clusters. ii) Medium segments (23–26 residues) showed a distribution corresponding to an intermediate state of transitions. iii) Long segments (27–50 residues) showed a distribution converging on one huge cluster containing compact conformations with a smaller radius of gyration. This distribution reflects the protein structures' organization and protein domains' origin. Three major conformational components (radius of gyration, structural symmetry with respect to the N-terminal and C-terminal halves, and single-turn/two-turn structure) well define most of the segment universes. Furthermore, we identified several conformational components that were unique to each structural class. Those characteristics suggest that protein segment conformation is described by compositions of the three common structural variables with large contributions and specific structural variables with small contributions.

**Conclusion:**

The present results of the analyses of four protein structural classes show the universal role of three major components as segment conformational descriptors. The obtained perspectives of distribution changes related to the segment lengths using the three key components suggest both the adequacy and the possibility of further progress on the prediction strategies used in the recent *de novo *structure-prediction methods.

## Background

Vast amounts of three-dimensional (3D) protein data from structural genomic studies and other individual efforts have been added to our knowledge, thereby enhancing our understanding of protein structures. To date, only two extremes of protein structural data have been studied. One extreme includes local features of proteins: those of short protein segments, typically of 10 residues long or less. The other extreme includes global features of proteins: protein folds or structural domains.

Regarding the short protein segments, abundant research examples exist partly because of the existence of variations of methods to analyze the local features of proteins. Various measures, such as RMSDs after structural superposition [1-3], **C**_α_-**C**_α _atomic distances coupled with the torsion angles [4,5], dihedral angles [6], and so on have been used to define the conformational similarity of protein segments. Different clustering techniques, such as *k*-means clustering [7,8], hierarchical methods [9], competitive learning [6,10], and other methods [11], have been used to describe the organization of the segments' conformational space. The abundance of research results in this area is also partly attributable to various applications of the clustering results of the short segments. A set of representatives from the resulting clusters are often called structural building blocks (SBBs). Even when using different procedures, clustering resolutions of SBBs can be categorized into only a few levels depending mainly on their respective applications, such as structural modeling, verification, comparison, and prediction [6,12]. The most dominant cluster of the short segments, which is common in all studies, corresponds to α-helices, whereas the variability of β-strands is observed at the high-resolution clustering. Regarding global features of proteins, understanding of their organization and analysis of the protein-fold (or structural domain) space studies are progressing well.

As reviewed recently [13], both hierarchical and continuous aspects of fold space have been realized. Regarding hierarchical classification, widely used databases such as CATH [14] and SCOP [15] have been constructed. Other databases such as FSSP [16] and VAST [17] have been developed. They are based on continuous measurements of protein structural similarity. Several studies have provided insights into the nature of fold space. Holm and Sander first described the conformational distribution of protein folds in a *fold universe *with multi-dimensional scaling methods based on an all-on-all comparison using the Dali program [18]. Using the same measurement, Hou *et al*. [19] showed visual representations of the protein fold universe and identified three major components which characterize the fold space: secondary structure compositions, chain topologies, and the protein domain size.

Compared to these two extremes, limited surveys have been done on the conformational space of medium size segments between protein short segments and folds. Specifically, supersecondary structures such as α-hairpin, βαβ-unit, and β-hairpin are typical structural motifs of medium size; those motifs have been analyzed. For example, Salem *et al*. reported that most superfolds contain a higher proportion of their α-helical or β-strand residues in one such supersecondary structure [20]. Szustakowski *et al*. built a dictionary of supersecondary structures [21]. Kurgan and Kedarisetti studied regularity among twilight zone protein structures at the level of the sequence segments that correspond to the secondary structure fragments of varying length [22]. However, the organization and statistical properties of the whole conformational space of medium-to-long segments remain unclear. Statistical and systematic analyses should be done on the 'segment universe' from short to long lengths to bridge this gap.

Our previous study identified structural clusters and visualized the uneven distribution of short segments in the conformational spaces of 6–22 residues, where known and novel secondary-structure motifs are distributed as isolated clusters [23]. The general features of the segment distribution were consistent for these lengths. However, the question we sought to answer is: Do spaces of long segments differ from those of short segments? In this study, we explore the relationships between the conformational distribution of segments and their length: 10–50 residues, thereby providing a global view of a 'segment universe' and showing critical dual changes (i.e. dual transitions) of the distribution shape in the conformational space of short to long segments. The critical changes might reflect changes of the protein structures' organization. Therefore, the present results suggest the adequacy and the possibility of further progress of the hierarchical treatment used in the recent *de novo *structure prediction methods. Furthermore, by comparing conformational components among structural classes (i.e., all-α, all-β, α/β, and α+β), we demonstrate the specificity and generality of protein fold classes.

## Results

### Transitions of segment distribution: short, medium, and long segments

The *coverage *of segments in cluster(s) was calculated as described below. A densely populated region in the 3D principal component analysis (PCA) space was defined as a cluster [23]. Given a density threshold, the segments are classifiable into two groups: those in regions of a density larger than the threshold and those outside the regions. The coverage of segments in clusters is defined as a ratio of the segments in the regions to all the segments.

**Figure 1:**
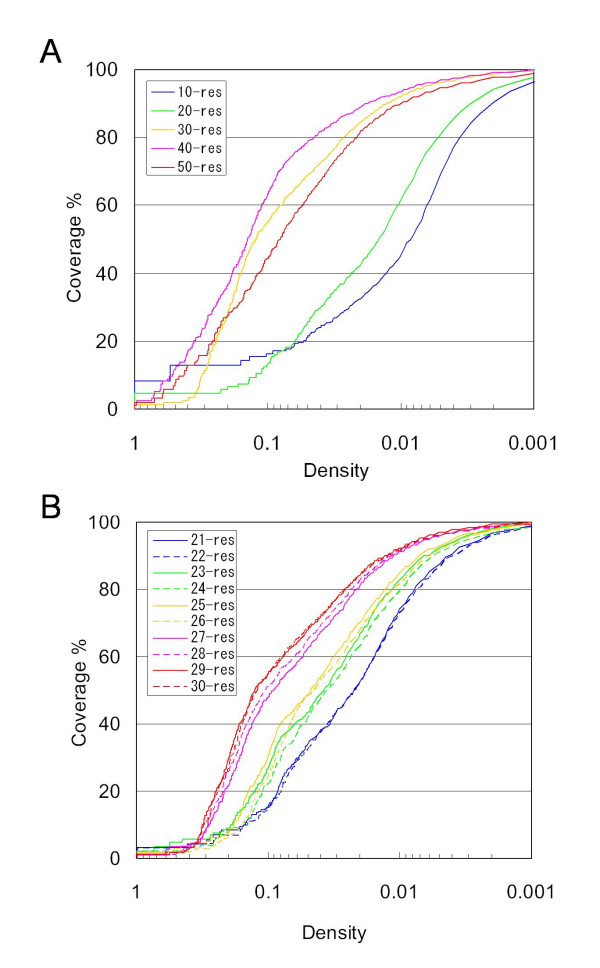
**Coverage versus the density threshold**. Coverage of segments in clusters versus the density threshold for segment lengths of 10, 20, 30, 40, and 50 residues (A), and those of 21–30 residues (B). Density is presented on a logarithmic scale.

Figure 1a portrays the coverage of segments versus the density threshold for the conformational spaces of 10, 20, 30, 40, and 50 residue lengths. The coverage curves exhibited a transition from concave shapes for short lengths (10 and 20 residues long) to convex ones for long lengths (30, 40, and 50 residues long). Notably, the differences of coverage at a density of 0.2 or less show a transition between the short and long segments. For instance, at a density of 0.1, the coverage is only 16.3% for 10 residues, although the coverage is greater than 50% for 30 residues. In addition, at a density of 0.01, the coverage for 10 residues is 45.6%, although coverage for 30 residues is 91.9%. These quantitatively indicate that the density gradient of the conformational space changes markedly with segment elongation.

**Figure 2:**
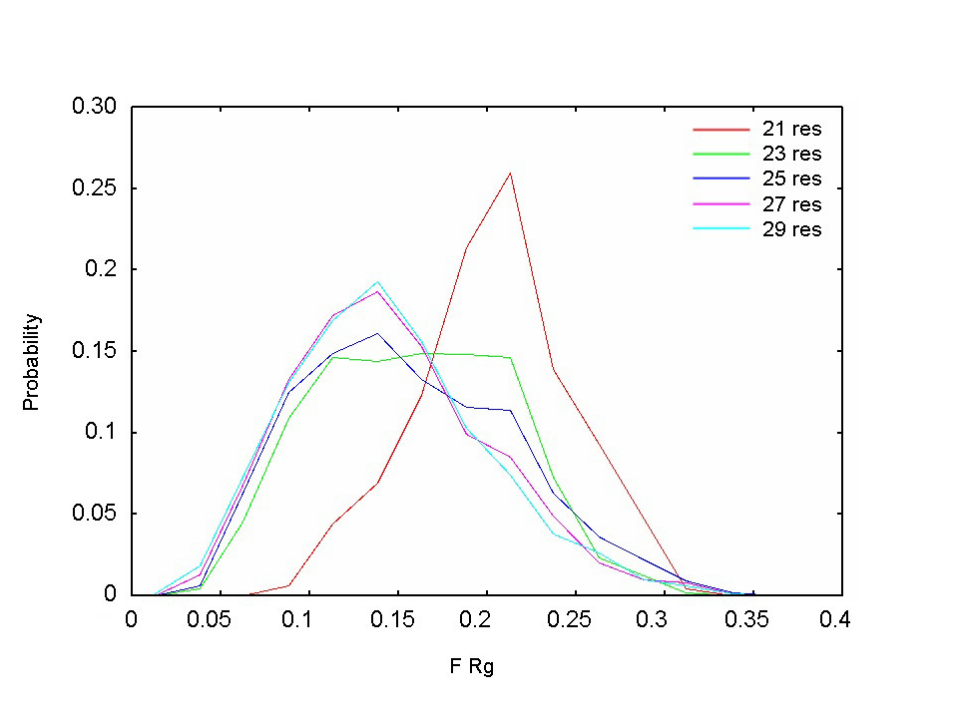
**Probability density function of the *F_Rg *score**. Distribution of *F_Rg *score of segments in the region with density from 0.10 (magenta in Fig. 3) to 0.35 (red). Distributions for segment lengths of 21, 23, 25, 27, and 29 residues are shown.

**Figure 3:**
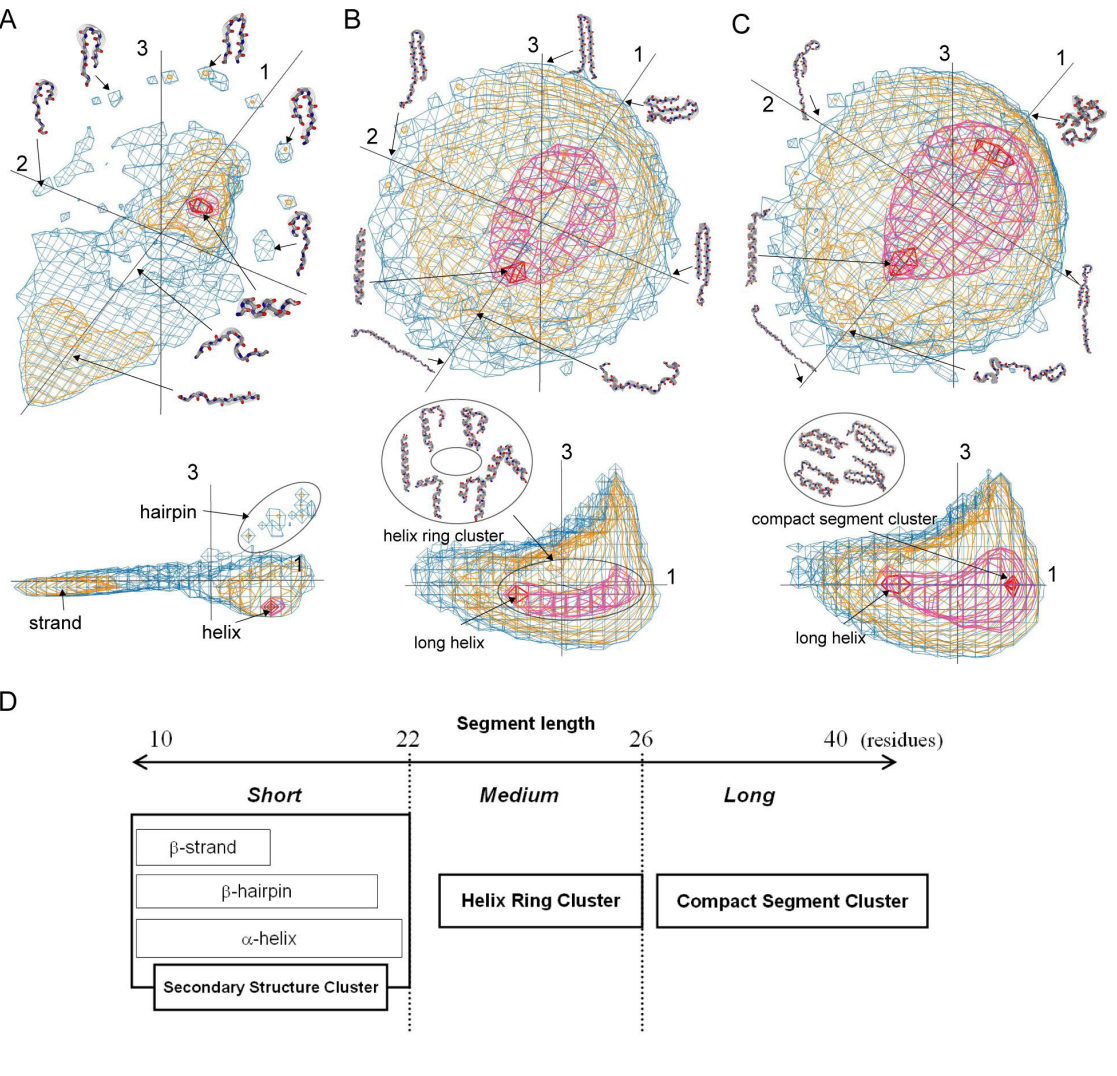
**3D representation of segment universes**. A 3D representation of short (A, 10 residues), medium (B, 26 residues), and long (C, 30 residues) segment universes. The 3D representations were generated using the first three *PC*^*all *^axes. They were expressed by four iso-density contours: 0.005 (blue), 0.01 (orange), 0.1 (magenta), and 0.35 (red). The PC axis numbers (1, 2, and 3) are given near the axes. Front and side views of images are shown, respectively, in the upper and lower areas of the figure. D: Schematic diagram of the relationship between properties of the cluster(s) in the segment universe and segment length. The properties of the cluster(s) in the segment conformational space changed according to increased segment length.

Further analyses of the coverage graphs between the short and long segments were meaningful to discover the boundaries of distribution changes. Figure 1b shows coverage curves for lengths of 21–30 residues. The dual and critical transitions, with an intermediate phase for segment lengths of 23–26 residues, can be recognized clearly, as presented in Fig. 1b. The transitions at intermediate length are also characterized by the distributional alteration of the radius of gyration of segments in the populated region with density of 0.10–0.35 (Fig. 2). To adjust the effect of different segment lengths, we defined here the relative score (*F_Rg*) of the radius of gyration for a segment as (*Rg*_(*i*,*j*) _- Min *Rg*_(*j*)_)/(Max *Rg*_(*j*) _- Min *Rg*_(*j*)_), where *Rg*_(*i*,*j*) _denotes the radius of gyration of a segment *i *with length *j*, and where Max *Rg*_(*j*) _and Min *Rg*_(*j*) _represent the maximal and minimal radius of gyration of the entire segment dataset with length *j*. Based on these observations, the segment length is categorized into the following three groups: short (10–22 residues), medium (23–26 residues), and long (27–50 residues). We were able to show that changes in the density gradient are associated with distributional alterations in the segment universe in subsequent analyses of visualizing the 3D PCA space. In fact, the difference in the coverage between lengths of 10 and 30 residues was attributable to the increase in the volume for the most populated region, as discussed below. The typical global images of segment universes from the three categories are depicted in Fig. 3d. The segment universes here were generated by the first three principal components derived from the entire segment dataset: *PC*^*all*^*1*, *PC*^*all*^*2*, and *PC*^*all*^*3 *(see *Methods*).

### Short length (10–22 residues long)

The conformational space of short segments showed a distribution with an extreme density gradient that originated from secondary structure clusters: α-helix and β-strand clusters were discriminated using a density of 0.01 (shown in orange in Fig. 3a). Between the lengths of 10 and 20 residues, spatial arrangements of the segment distribution, especially for α-helical, β-strand, and β-hairpin clusters, were conserved in short conformational spaces. The highly populated core of the α-helix cluster exhibited a density of 0.1 (shown as magenta in Fig. 3a), consisting of completed α-helical segments. The surrounding area of the central region consisted of various types of helical conformations including helix-capping motifs [12]. The central region of the β-strand cluster consisted of fully extended segments that originated mainly from β-sheets and loop regions. The β-hairpin conformations were separated into several clusters at a density of 0.005. Then they were discriminated using the coordinate *c*_2 _along *PC*^*all*^*2 *(see *Methods *for the definition of *c*_2_). The β-hairpin clusters showed a symmetrical relationship related to the N-terminal and C-terminal halves. They were arranged symmetrically around an edge of segment universes of short length.

### Medium length (23–26 residues long)

**Figure 4:**
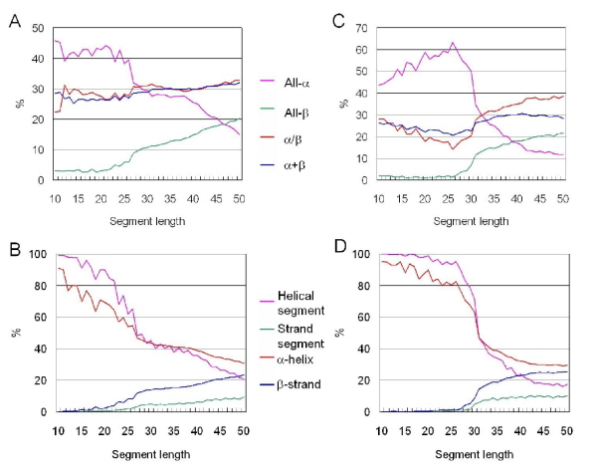
**Relationships between segment properties and their length in the populated regions**. Upper figures show that the percentages of structural classes from which segments in the clusters with density of 0.10–0.35 (A) and 0.35–1.0 (C) were derived. Lower figures show the percentages of structural properties of the segments in the clusters with density of 0.10–0.35 (B) and 0.35–1.0 (D). The magenta and green lines in lower figures represent the percentage of helical and strand segments in the cluster. A helical segment is defined as the segment with the rate of α-helical residues >= 0.5. A strand segment is defined as the segment with the rate of β-strand residues >= 0.5. The percentages of helix (H) and sheet (E), that are determined using the DSSP program [40], are also represented by the red and blue lines, respectively.

The segment distribution for medium lengths differed from that for short lengths. The distributional change from short to medium lengths is characterized using a diminishing β-strand cluster and a growing α-helix cluster. The overall distribution was shortened in the direction of *PC*^*all*^*1*, and enlarged in the direction of *PC*^*all*^*2 *and *PC*^*all*^*3*. In the segment universe of 26 residues, the α-helix cluster was discriminated using a density of 0.1 (magenta in Fig. 3b). Interestingly, the shape of the α-helix cluster was a ring (designated as a *helix ring cluster*). The helix ring cluster that is specific to the medium-length universe consisted not only of the extended α-helices but also of various α-helical conformations, as presented in the inset of Fig. 3b. This cluster included conformations that had originated mainly from all-α, α/β, and α +β proteins (Fig. 4a). The average content of the α-helical residues per segment in the helix ring cluster was about 50% (Fig. 4b); 24.9% of all segments were included within the helix ring cluster. The long-α-helical segments, whose conformation was not compact, were located near the origin of the conformational space (red in Fig. 3b). In contrast, the α-hairpin conformations with a small radius of gyration were located on the opposite side of the position on *PC*^*all*^*1*. The various α-hairpin conformations with the different turn positions were located symmetrically along *PC*^*all*^*2*. For medium lengths, the β-strand clusters were diminished because long extended β-strands are rarely found in proteins. The β-hairpin conformations were located symmetrically along *PC*^*all*^*2*, although the cluster separation of β-hairpins was not clear in medium lengths.

### Long length (27–50 residues long)

Conformational spaces for the long lengths were further shortened in the direction of *PC*^*all*^*1 *and enlarged in that of *PC*^*all*^*3*. The segment distribution converged on a large populated region that exhibited a density of 0.1 (magenta in Fig. 3c) in the conformational space. With a length of 30 residues, there were two clusters consisting of compact segments and long α-helical segments, respectively, with densities of 0.35 (red in Fig. 3c) in the populated region. The emergence of the compact-segment cluster was attributable to an increase in various types of segments with a small radius of gyration (see inset of Fig. 3c). Various types of conformations are mixed up in the compact-segment cluster. The α-hairpins are derived mainly from all-α proteins. The compact β-sheet structures are derived mainly from all-β proteins. Compact conformations of other types are derived from α/β and α +β proteins (Fig. 4c). About 2% of all segments were included in the compact-segment cluster for 27-residue length. In contrast, long α-helical segments with a large radius of gyration were located on the opposite side of the cluster of the compact segments along the *PC*^*all*^*1 *axis. For lengths greater than 30 residues, the proportion of the conformations with a small radius of gyration in the compact-segment cluster increased rapidly to around 14% for 50-residue lengths. Those conformations were derived from various folds (Fig. 4c). The supersecondary structures, such as βαβ units and β-sheets, were included in the compact-segment cluster (Fig. 4d).

### Contribution ratios of principal axes

**Figure 5:**
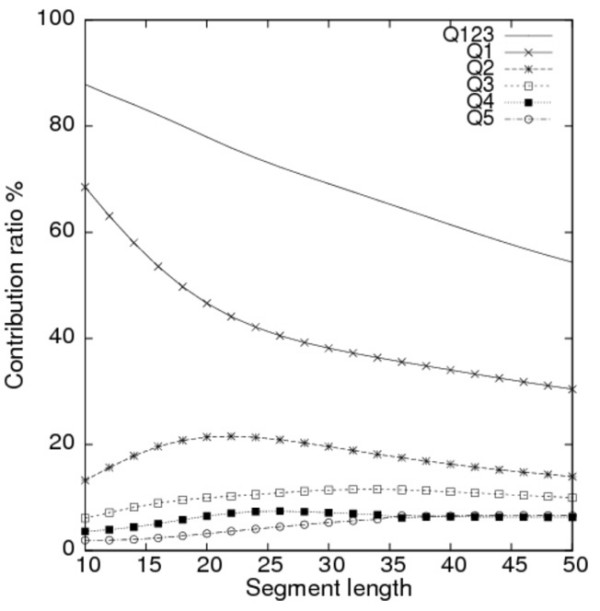
**Contribution ratios for the PC axes**. Contribution ratios for the first five PC axes for segment lengths of 10–50 residues are shown here. The cumulative contribution ratio for the first three PC axes (*Q*_123_, solid line with no symbol) and the individual contribution ratios of *PC*^*all*^*1 *(*Q*_1_, crosses), *PC*^*all*^*2 *(*Q*_2_, asterisks), *PC*^*all*^*3 *(*Q*_3_, squares), *PC*^*all*^*4 *(*Q*_4_, filled squares), and *PC*^*all*^*5 *(*Q*_5_, circles) are shown.

Distributional alterations were observed associated with the changes of segment length. For principal component analyses, the contribution ratios (see *Methods *for the contribution ratios) of the principal components (i.e. PC axes) to the entire distribution indicate how well the PC axes can cover the variation in the original data. Figure 5 portrays contribution ratios of the first five PC axes (*PC*^*all*^*1 *– *PC*^*all*^*5*) for segment lengths of 10–50 residues. Even with a length of 43 residues, the cumulative contribution ratio of the first three PC axes, *Q*_123 _(= *Q*_1 _+ *Q*_2 _+ *Q*_3_), was greater than 60%, although *Q*_123 _decreased constantly with increased segment length. Each of *Q*_4 _and *Q*_5 _was always less than 8%. The contribution ratios for higher-order PC axes than *PC*^*all*^5 did not exceed 5% for the examined segment lengths. Therefore, it is sufficient to use only the first three PC axes (or the first five PC axes occasionally) to explain the original structural variation.

With respect to the individual contribution ratios (*Q*_1_-*Q*_3_) of the first three PC axes, *Q*_1 _was overwhelmingly higher than those of the other PC axes up to 50-residue length (Fig. 5), which indicates that *PC*^*all*^*1 *is a meaningful and fundamental descriptor for segment conformation. Actually, *Q*_1 _decreased rapidly, and *Q*_2 _increased in the short segment lengths (i.e. 10–22 residues). Thereafter, both *Q*_1 _and *Q*_2 _decreased slowly. In addition, *Q*_3 _increased gradually with lengths up to 33 residues, with a maximum value of 11.5%.

### Investigation of structural properties of conformational axes

**Figure 6:**
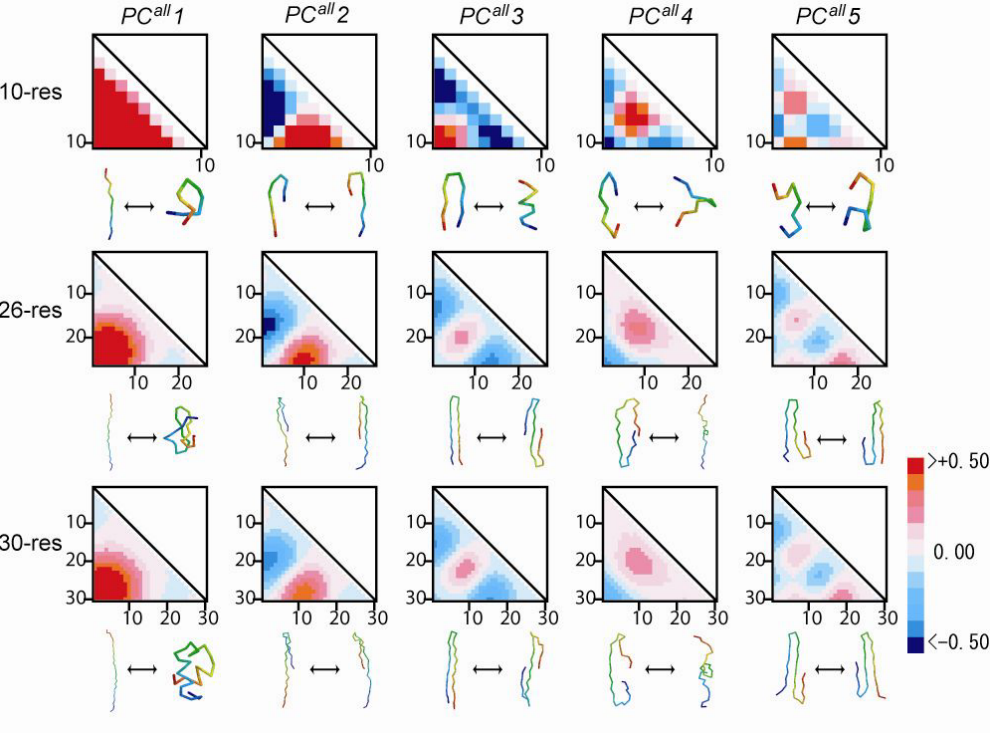
**Distance matrices for the PC axes**. Distance maps express eigenvectors for the first five PC axes for 10, 26, and 30 residue lengths. Each map shows deviations of **C**_α_-**C**_α _distances along each eigenvector from the mean **C**_α_-**C**_α _distances. The scale bar indicates the relative deviation. The color indicates whether in this particular mode the distances are increasing (red) with respect to the mean value or decreasing (blue). The eigenvector values are scaled by the square root of eigenvalue of the PC axis *k*, *λ*_*k*_^1/2^. Residue numbers are displayed with horizontal and vertical sides of each triangle map. Two segment conformations were picked up from both ends on each PC axis; they are displayed under each triangle map.

**Figure 7:**
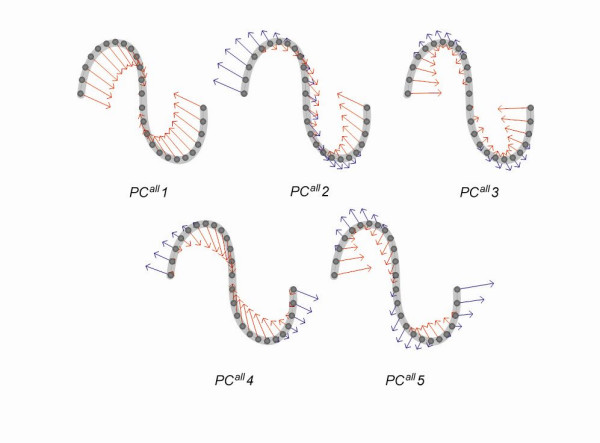
**Visualization of collective variables for the first five PC axes of 26 residue lengths**. Collective variables (eigenvectors) for the first five PC axes of 26 residue lengths are visualized by the vectors onto **C**_α _atoms of a segment. The vector for the **C**_α _atom of residue *i *is calculated as *r*_*i *_= Σ_*i *≠ *j*_***q***_*ij*_*e*^*vk*^_*ij*_*λ*_*k*_^1/2^, where ***q***_*ij *_is the vector from residue *i *to *j*, *e*^*vk*^_*ij *_is the element of eigenvector of the PC axis *k *(***v***_*k*_) corresponding to the **C**_α_-**C**_α _pair between residue *i *and *j*, and *λ*_*k *_is the eigenvalue of the PC axis *k*. Positive (blue) and negative values (red) are shown for elements of eigenvectors. The reference segment used in this figure is designed to clarify the difference between structural variables.

An eigenvector was analyzed for each PC axis with a triangle map to elucidate the physical and conformational meaning of the PC axes of the conformational space of the short to long segments. The eigenvector can be regarded as a collective variable to describe the segment conformation. Figure 6 shows triangle maps of the first five PC axes (*PC*^*all*^*1 *– *PC*^*all*^*5*) for short (10 residues), medium (26 residues), and long segments (30 residues). The triangle map clearly portrays residue pairs, with large or small deviations of **C**_α_-**C**_α _distances along each PC axis from the average distance <*q*_*i*_>. In the triangle map, positive (red) and negative (blue) areas correspond to residue pairs with mutually inverse deviations. The patterns of red and blue areas are conserved in the universes of short to long segments, indicating that conformational deviations related to the PC axes are conserved among the universes. Figure 7 depicts the conformational changes along the PC axis using colored arrows.

**Figure 8:**
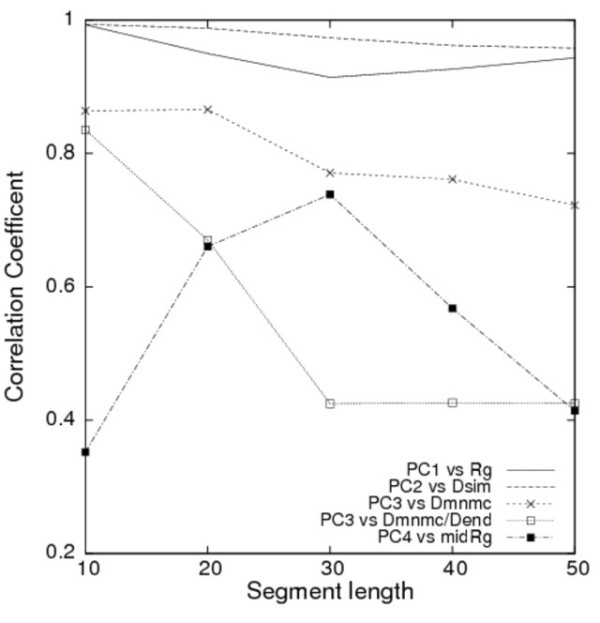
**Correlation coefficients between conformational deviation along each PC axis and physical indicator**. The radius of gyration (*Rg*) for *PC*^*all*^*1*, structural symmetry related to the N-terminal and C-terminal halves (*D*_*sim*_) for *PC*^*all*^*2*, a simple indicator of β-hairpin (*D*_*mn*+*mc*_) and *D*_*mn*+*mc*_/end-to-end distance (*D*_*end*_) for *PC*^*all*^*3*, and the radius of gyration (_*mid*_*Rg*) around the midpoint of the segment for *PC*^*all*^*4 *were used in these analyses. Correlation coefficients were calculated at every 10 residues of 10–50 residues.

Actually, *PC*^*all*^*1 *corresponds to the change of the radius of gyration (*Rg*). The triangle map for *PC*^*all*^*1 *has only one positive area, shown as red in Fig. 6, which is located near the residue pairs at the N-terminal and C-terminal sides. This single area indicates that the distant residue pairs in the sequence have a larger conformational deviation along *PC*^*all*^*1*. The correlation coefficient of the conformational deviation along *PC*^*all*^*1 *with *Rg *was greater than 0.9 in segment lengths of 10–50 residues (Fig. 8). The arrows in Fig. 7 point to the center of the segment, which indicates clearly that the conformational changes along *PC*^*all*^*1 *are involved with expansions or compressions of the conformation. For short lengths, *PC*^*all*^*1 *also shows a strong correlation with the changes of the segment end-to-end distance (*D*_*end*_), which is defined as the **C**_α_-**C**_α _distance between the first and last residues of segments. Correlation between *PC*^*all*^*1 *and *D*_*end *_slowly weakened with increased segment length: 0.91 for 10 residues, 0.79 for 26 residues, and 0.77 for 30 residues.

The *PC*^*all*^*2 *correlates to a degree of structural symmetry (*D*_*sym*_) of a segment with respect to the N-terminal and C-terminal halves. The *D*_*sym *_is defined as follows: Given a distance matrix for a segment, where element (*i*,*j*) is the distance (denoted as *r*_*ij*_) between **C**_α _atoms of residue *i *and *j*. Then, the degree of structural symmetry is defined as the sum of the squared differences of symmetric elements in a distance matrix for a segment: *D*_*sym *_= Σ_1 ≤ i < j ≤ n _(*r*_*ij *_- *r*_*n*-(*j*-1)*n*-(*i*-1)_)^2^, where *n *is the segment length. The triangle map for *PC*^*all*^*2 *was separated into one positive area (red) and one negative area (blue). The correlation coefficient of the conformational deviation along *PC*^*all*^*2 *with structural symmetry, *D*_*sym *_was greater than 0.90 in the segment lengths of 10–50 residues (Fig. 8). Both conformations displayed mirrored symmetry about a plane constructed by *PC*^*all*^*1 *and *PC*^*all*^*3 *when two conformations were picked from opposite positions along *PC*^*all*^*2*. The segment conformations picked up along *PC*^*all*^*2 *are shown in Figs. 3a–3c.

The *PC*^*all*^*3 *correlated with a physical indicator that describes a conformational transition between structures with one turn and ones with two turns (*PC*^*all*^*3 *in Fig. 6). The picked conformations along *PC*^*all*^*3 *indicate that segregation of a β-hairpin structure exists along with conformational changes by *PC*^*all*^*3*. We defined the physical indicator (*D*_*mn*+*mc*_) of the β-hairpin formation: *D*_*mn*+*mc *_is the sum of the norms of two vectors, which were generated by the middle point of the segment for both the N-terminal and C-terminal residues: *D*_*mn*+*mc *_= $\left|\vec{d_{mn}}+\vec{d_{mc}}\right|$, where $\vec{d_{mn}}$ and $\vec{d_{mc}}$ respectively denote the vectors from the midpoint to the N-terminal and C-terminal residues of the segment. Good correlation was found between *PC*^*all*^*3 *and *D*_*mn*+*mc *_(Fig. 8). The correlation coefficient was greater than 0.7 for the 10–50 residues. The triangle map of *PC*^*all*^*3 *indicated a separation of one positive area (red) and two negative areas (blue). It is noteworthy that the triangle map of *PC*^*all*^*3 *for short segments differed slightly from those of medium and long segments. A positive area is visible near the residue pair of the N-terminal and C-terminal in the short map, suggesting that *PC*^*all*^*3 *has a (negative) correlation with *D*_*end*_. For medium and long lengths, the positive area was close to the center of the triangle map. Therefore, the correlation between *PC*^*all*^*3 *and *D*_*mn*+*mc*_/*D*_*end *_was necessarily smaller in medium and long lengths.

The triangle map of *PC*^*all*^*4 *had one negative area and one positive area. The positive area, located at the map center, suggests that *PC*^*all*^*4 *is correlated with the radius of gyration (_*mid*_*Rg*) of the middle region of the segment – except for both the N-terminal and C-terminal quarter portions – in the medium and long segments. The respective correlation coefficients for the 26 and 30 residue lengths were 0.73 and 0.72. The *PC*^*all*^*4 *also has a weak (negative) correlation with *D*_*end*_. The respective correlation coefficients between *PC*^*all*^*4 *and *D*_*end *_for the 26 and 30 residue lengths were -0.45 and -0.42.

We identified no simple physical indicator for conformational changes along *PC*^*all*^*5*. However, visual inspection from conformations picked along *PC*^*all*^*5 *suggests that *PC*^*all*^*5 *is a conformational axis that represents segregated β-sheet structures. Conformations picked up from both ends on *PC*^*all*^*5 *are depicted in Fig. 6. In the triangle map for *PC*^*all*^*5*, two positive and two negative areas exist along the diagonal line, which might indicate that *PC*^*all*^*5 *segregates segment conformations with double turns. The *PC*^*β*^*5 *contribution ratio, which was derived from all-β proteins, was higher than that derived from other structural classes, which suggests that *PC5 *is important for describing the structural variation of β-structures.

### Segment universes derived from different structural classes

The segment universes described above are those derived from proteins of the four structural classes. Therefore, decomposition of the universe into four classes is helpful to evaluate the influence of each structural class on the segment universe. To this end, a segment universe was constructed for each structural class separately, and compared the PC axes derived from each universe with those of all segments (i.e., *PC*^*all*^*1-PC*^*all*^*3*). The first three largest eigenvectors of each structural class were also compared respectively with *PC*^*all*^*1*, *PC*^*all*^*2*, and *PC*^*all*^*3 *to elucidate the structural properties of PC axes derived from each universe.

**Figure 9:**
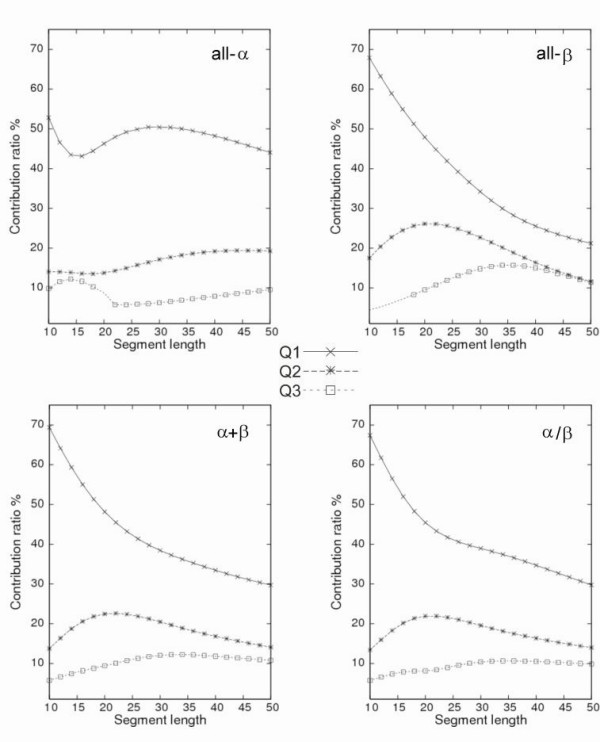
**Contribution ratios for the PC axes for each structural class**. Contribution ratios (*Q*_1_, crosses; *Q*_2_, asterisks; and *Q*_3_, squares) of *PC*^*x*^*1*-*PC*^*x*^*3 *vs. segment lengths of 10–50 residues for each structural class, where *x *= α, β, α+β, or α/β. The correlation coefficient between *PC*^*x*^_*i *_and *PC*^*all*^_*i *_(*i *= 1, 2, 3) is 0.7 or less if no mark is present at a segment length. For the all-β class, no axis exhibited a correlation coefficient greater than 0.7 up to *PC*^*all*^*3 *for segment lengths of 10–16 residues.

Figure 9 depicts the contribution ratios of the first three PC axes, *PC*^*x*^*1 *-*PC*^*x*^*3 *(*x *= α, β, α +β, or α/β), in each structural class. The marks on the curves in Fig. 9 indicate that the correlation coefficient (***v***^*x*^_*i*_·***v***^*all*^_*i*_) between *PC*^*x*^*1 *-*PC*^*x*^*3 *and *PC*^*all*^*1 *-*PC*^*all*^*3 *(i.e., *i *= 1, 2, 3) is greater than 0.7, which was used here as a threshold of conservation of structural properties. The properties of the first two PC axes corresponding to the *PC*^*all*^*1 *and *PC*^*all*^*2 *were highly conserved in all four structural classes. The characteristics of *PC*^*all*^3 were also conserved in all four structural classes, although exceptions were apparent for the 20-residue-long and 10–16-residue-long all-α and all-β classes. Therefore, it is confirmed that the first three PC axes (*Rg*, symmetry, and one/two turn(s)) are important in almost all cases to describe the conformation of segments embedded in globular proteins.

However, the curves for the contribution ratios of both all-α and all-β classes (see two panels of Fig. 9) differ clearly from those of *PC*^*all*^*1 *– *PC*^*all*^*3 *(i.e. *Q*_1 _– *Q*_3 _in Fig. 5). The *Q*^*α*^_1_, contribution ratio was always higher than 40%, which indicates that the distribution of the all-α segments has a large deviation with respect to *Rg*. In contrast, the *Q*^*β*^_1 _contribution ratio decreased rapidly with increasing segment length. The value of *Q*^*α*^_2 _increased moderately with increasing segment length. In contrast, the *Q*^*β*^_2 _had a maximum value greater than 20% at a length of 22 residues. This rapid increase of *Q*^*β*^_2 _might reflect a typical feature for β-sheet conformations. For *PC3*, the curves for the contribution ratios of the all-α and all-β classes also mutually differed. Although *Q*^*β*^_3 _peaked at a length of 35 residues, *Q*^*α*^_3 _peaked with a short length, which indicates that the structural variable based on *PC*^*all*^*3 *is important for β-segments longer than 30 residues. In contrast, the behaviors of the contribution ratios for both α+β and α/β classes along with the segment length resembled each other. They were also similar to *Q*_1_-*Q*_3 _in Fig. 5 because those structural classes are mixtures of α-helices and β-sheets.

**Figure 10:**
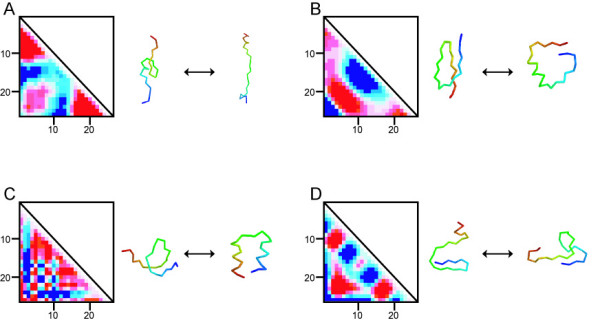
**Examples of class-specific conformational axes**. Conformational axes specific to structural class for 26-residue segments are shown. Eigenvector maps and conformations picked up from both ends on each PC axis are shown: A for *PC*^*α*^*8*, B for *PC*^*α/β*^*8*, C for *PC*^*α*^*10*, and D for *PC*^*β*^*10*. The color contrast of the maps is enhanced to aid viewing. The anti-phase of the blue color is shown in red. Residue numbers are displayed with horizontal and vertical sides of each triangle map. The respective contribution ratios of *PC*^*α *^*8*, *PC*^*α/β*^*8*, *PC*^*α*^*10*, and *PC*^*β*^*10 *were 1.3%, 1.4%, 0.9%, and 0.7%.

Subsequently, PC axes that were specific for each structural class were examined. For this analysis, the PC axis was defined as a "class-specific" one when a PC axis from a structural class showed no similarity with the first 20 PC axes from the other three structural classes (see *Methods*). The first 10 PC axes of each class were investigated for the short (10 residues), medium (26 residues), and long (30 residues) segments. Ten class-specific conformational axes were identified and consisted of one (*PC*^*β*^*10*) for the short length, eight for the medium, and one (*PC*^*α*^*8*) for the long. The eight class-specific axes for the medium-length segments are *PC*^*α*^*5*, *PC*^*α*^*8*, and *PC*^*α*^*10 *for all-α, *PC*^*β*^*10 *for all-β, *PC*^*α+β*^*9 *and *PC*^*α+β*^*10 *for α+β, and *PC*^*α/β*^*8 *and *PC*^*α/β*^*10 *for α/β. Four examples out of eight are depicted in Fig. 10. A clear correlation of these PC axes is difficult to discern according to simple physical or structural quantities. Figure 10a shows that the *PC*^*α*^*8 *describes a structural change of three (both ends and the middle portion) parts of α-segments. The *PC*^*α/β*^*8 *is related to βαβ motifs, which is the most fundamental structural unit for α/β proteins.

## Discussion

Investigation of the protein segment universe is an important subject for bioinformatics. Results of this study show that the segment universe can be categorized naturally into three regimes: short, medium, and long. A main finding of this study is that the three regimes are clearly demarcated by critical changes in the shape of the segment distribution in the conformational space. Preceding studies demonstrated that the average length of α-helix is 14 residues [24] and that for β-strand is five residues [25]. Results of the present study show that transitional segment lengths (22 and 26 residues long) do not coincide with these average lengths. Therefore, a single secondary structure element does not characterize the shape of the segment distribution. The appearance of the medium length regime segregates the segment fold universe into three. The combination of secondary-structure elements is important to characterize not only the medium-length segment universe but also the entire segment fold universe.

Meanwhile, loops, which make up 30% of the protein structures [26], are also expected to take a larger role to form some unique conformations by connecting secondary-structure elements in the medium to the long-length segment universe than short one. The segments in the cluster of the medium to long-length universe tend to contain more loop regions than those of the short segment universe, as shown in Figs. 4b and 4d, and have a wider variety of origins (Figs. 4a and 4c). For example, the segments in the cluster with density of 0.35–1.0 of the universe of 30 residues length are derived from 461 proteins out of all 600 representatives used for this study (see Additional File 1). Longer loops that possess extended conformations are located on the opposite side of the compact-segment cluster along *PC*^*all*^*1 *in the medium to long segment universe (Figs. 3b and 3c). Instead of discrete clusters, they appear to constitute a rather continuous distribution. Some analyses examine short loops with respect to their completeness [27,28] and elaborate classification [26,29]. In the analysis of short segments, our method also captured some loop conformation classes, such as joint loops connecting two helices, and exposed and extended loops participated in protein-protein interactions [23].

A natural boundary was identified, in this study, between the peptide-like and protein-like distributions between the lengths of 23 and 26 residues using actual conformations of protein segments. This observation with respect to the boundary is consistent with the results described by Shen et al. [30], even though they used a sphere-packing model to estimate a minimal domain size of about 20 residues. A recent study by Sawada and Honda [31] also identified a boundary at 10–20 residue length by calculating the structural diversity of segments. They discretized the conformational space using a single-pass clustering method. In contrast, we observed the density distribution to uncover differences of conformational space between short and long segments. The segment conformational space for lengths of 10–22 residues provided a distribution with an extreme density gradient towards the secondary structure, such as the α-helix, β-strand, and β-hairpin clusters, which are expected to belong to the peptide-like conformational regime. This conformational variation reflects that short segments embedded in globular proteins are mainly stabilized by the physicochemical property of the peptide. On the other hand, the segment conformational spaces for lengths of 27 residues or more have a distribution that is dominated by compact segments, which suggests a protein-like distribution (protein-like conformational regime). This distribution arises from the hydrophobic effect imparted by the solvent molecules, which is of great importance for structural stability in long segments derived from globular proteins. If this is the case, our observations support the *de novo *structure prediction methods, so-called fragment assembling methods, that have been developed recently [32-35]. These approaches are usually based on the prediction of local segment conformations followed by assembly of segments, and are generally used to separate criteria at each step; sequence similarity or secondary structural propensity for the prediction of segment conformations, and non-local energy terms for the assembling step. These strategies used in the *de novo *prediction methods seems to be consistent with the results shown here. Results of our analyses clearly show such a hierarchical organization of protein structures, and indicate that preparing segment libraries up to around 20 residues long would be helpful for such methods.

These results indicate that the structural meanings for the conformational axes (i.e., the radius of gyration for *PC*^*all*^*1*, structural symmetry related to the N-terminal and C-terminal halves for *PC*^*all*^*2*, and a single-turn/two-turn structure for *PC*^*all*^*3*) are conserved in the different lengths and structural classes. This fact suggests that these conformational components are key structural variables for protein segments. On the other hand, when conformational axes among the four structural classes were compared, we were able to identify several conformational axes that were specific to each structural class, especially in the medium length range. In fact, a distribution change for medium lengths was observed, involving an increase in compact segments. Those segments included supersecondary structures such as α-hairpins, parts of the β-sheets, and βαβ units. These results might be related to the specificity of the structural class or fold of the contents of supersecondary structures [20]. Typical supersecondary structural motifs, α-hairpin, β-hairpin, and βαβ are, respectively, the basic structural units for the all-α, all-β, and α/β proteins. These motifs are often shared within the structural classes. Therefore, the contribution ratios observed for the class-specific conformational axes were high. Class-specific conformational axes were rarely observed in short and long lengths, probably because short segments are too nonspecific and are often shared over different structural classes; long segments are too specific and have very low contribution ratios for conformational axes that are specific for each structural class.

The currently found class-specific conformational axes provide a hint to solve a difficulty in classifying diverse sets of protein structures. Both α/β and α+β classes are known to show a substantial overlap. In the CATH classification, α/β and α+β classes are treated as one structural class as α-β class. Classifying α/β and α+β proteins is sometimes a difficult problem, although several classification [19,36,37] and also prediction [38,39] schemes have been proposed. The present study showed that α/β and α+β classes have similar characteristics of universes, and also have unique ones at the same time. For example, our results show that *PC*^*α*/*β*^*8*, whose contribution ratio was 1.4%, was associated only with the βαβ motif. In the α+β class, no axis was strongly correlated with *PC*^*α*/*β*^*8 *(see Additional File 2), which is a clear example of the difference in structural variables between α+β and α/β classes originating from class-specific supersecondary structures. Consequently, projecting segments onto a conformational subspace using the axis *PC*^*α*/*β*^*8 *could be useful for objectively dividing protein domains of α-β class into α/β and α+β classes. A considerable localization of segments derived from α/β proteins in a PCA subspace is observed (see Additional Files 3 and 4).

An effective method must be developed for conformational sampling for *de novo *prediction methods. The resulting structural variables analyzed in this study would be helpful for additional progress in *de novo *structure prediction. For example, testing the distribution of segments or models in terms of the degree of symmetry using the descriptor (*D*_*sym*_) might be useful to verify the completeness of sampling of the conformational space. Using a filtering threshold or function (generally used in fragment assembling methods for selecting proper models) that is tolerant of the radius of gyration might be useful for improving the prediction of all-α proteins because the contribution ratio, *Q*^α^_1_, of *PC*^α ^*1 *corresponding to the radius of gyration (*Rg*) is larger than those of the other structural classes in the medium and long segments. Consequently, projecting segments of models onto a conformational subspace constructed by **PC**^*x *^(where *x *= α, β, α/β, α+β, or all) axes might be helpful for filtering out models and assigning a protein to a structural class.

## Conclusion

In this study, the dual critical transitions in the protein segment universe from short to long length are shown. Our observations are related to the transitions proposed by the significance of two-stage treatment in *de novo *structure prediction. Considering the hierarchical organization of a protein segment universe that we have shown, we suggest the efficacy of using the evaluation functions that is secondary-structure-directed for sampling local structures less than 23 residues long. We also suggest the suitability of evaluating protein-like features of models using another function (e.g. *Rg*) for longer segments. Changing the criteria of filtering for each structural class will enhance the effectiveness of the conformation sampling process. Through these analyses, we have demonstrated that our clustering methodology is useful to identify a distinctive distribution shift of conformational space between short and long segments and that distribution changes depend on structural classes.

## Methods

### Preparing the segment libraries

One representative from each fold group of the SCOP database (ver. 1.63) [15] was chosen to obtain a segment library without a bias of usage of the folds. The representatives cover the four major structural classes (all-α, all-β, α+β, and α/β), because we are interested in and specifically examine characterization of the nature of segments embedded in usual size globular proteins. Small proteins of less than 50 residues and non-single chain proteins with less than 100 residues were excluded, as were membrane proteins. It is expected that those proteins possess different structural properties from those of usual size globular proteins and induce biased results. In all, 600 representatives were used for this study (all-α, 150; all-β, 116; α+β, 219; α/β, 115; see Additional File 5). Dividing the protein structures into segments with a sliding window by one residue along the sequence generated a segment library of arbitrary length. We prepared a segment library for each length of 10–50 residues to generate conformational spaces of short-to-long segments. In such cases, segments with incomplete coordinate data (e.g., having an unusual covalent-bond length or lacking main-chain atoms) were excluded. Furthermore, to elucidate differences among the conformational spaces derived from the four major structural classes, we generated a segment library for each class.

### Construction and visualization of conformational space

We previously reported a method for constructing and visualizing the conformational space of protein segments using principal component analysis based on intra-segment **C**_α_-**C**_α _atomic distances [23]. Briefly, atomic distances of all **C**_α_-**C**_α _pairs for each segment in a segment library of an arbitrary length were calculated first. A distance is designated as *q*_*i*_, where *i *is the index for the **C**_α_-**C**_α _pair, *i *= 1, ..., *n*(*n *- 1)/2, and *n *is the segment length, as expressed by the number of residues in the segment. Subsequently, a set of eigenvectors and eigenvalues were obtained by diagonalizing a variance-covariance matrix, **C**, that was calculated as **C**_*ij *_= (<(*q*_*i *_- <*q*_*i*_>)(*q*_*j *_- <*q*_*j*_>)> = <(*q*_*i*_*q*_*j *_- *q*_*i*_<*q*_*j*_>- <*q*_*i*_>*q*_*j *_+ <*q*_*i*_><*q*_*j*_>)> = <*q*_*i*_*q*_*j*_>- <*q*_*i*_><*q*_*j*_>- <*q*_*i*_><*q*_*j*_> + <*q*_*i*_><*q*_*j*_> =) <*q*_*i*_*q*_*j*_>- <*q*_*i*_><*q*_*j*_>, where the average <...> is taken over the segments. Two equations, **C*v***_*i *_= *λ*_*i*_***v***_*i *_and ***v***_*i*_·***v***_*j *_= δ_*ij*_, are satisfied. Eigenvectors with larger eigenvalues are more important in the study of the conformational varieties of the segments. Eigenvalues are arranged in descending order: *λ*_*i *_> *λ*_*j *_if *i *<*j*. The contribution ratio of the *i*-th PCA element (i.e. the *i*-th eigenvector) to the whole conformational distribution is given as *Q*_*i *_= *λ*_*i*_/Σ_*k*_^*all *^*λ*_*k*_. The eigenvectors, which are called **PC^x^1**, **PC^x^2**, **PC^x^3**, ...etc., were used as conformational axes to construct a segment conformational space, a PCA space, in which *x *indicates a segment dataset: *x *= α, β, α/β, α +β, or all). The indicator "*x *= all" is given when conformational axes are generated by the whole segment dataset. The origin of the PCA space is set on the average **C**_α_-**C**_α _atomic distances: <***q***> = [<*q*_1_>, <*q*_2_>, <*q*_3_>, ..., <*q*_*n*_>]. This enables ready comparison of conformational distributions between constructed universes. Any position (i.e. any segment structure) in the PCA space can be expressed using a linear combination of eigenvectors as *c*_*k *_= Σ_*n*_^*all *^(***q ***- <***q***>)·***v***_*k *_*λ*_*k*_^1/2^, where *c*_*k *_is a coordinate (i.e. projection of ***q***) on the PC axis *k*. Using the first three eigenvectors (**PC^x^1**, **PC^x^2**, **PC^x^3**), a three-dimensional (3D) PCA space can be constructed.

We defined a vector, ***r***, to express the position of each segment in the 3D PCA space: ***r ***= [*c*_1_, *c*_2_, *c*_3_]. After projection of the segments on the 3D PCA space, the distribution of segments in the 3D PCA space was visualized using the following procedure. The 3D space was divided into *N *bins (total *N*^3 ^cubes). The bin size was defined as (max [*c*_1_] - min [*c*_1_])/*N*, where *N *= 36, and max [*c*_1_] and min [*c*_1_] respectively signify the maximum and minimum of the coordinates of the segments along the first principal component axis. The number (i.e. frequency) of segments detected in a cube represents the density (i.e. probability) of segments to be found in the cube. The density of each cube, ρ was normalized by the maximum density, ρ_max _among the cubes so that the maximal value of normalized density (we call this *density *in the text) is set to 1 (refer to eq. (3) in [23]). Four levels of contour surfaces (i.e. iso-density surfaces) were depicted to visualize the 3D PCA space. The density values for those surfaces were set respectively as 0.005, 0.01, 0.1, and 0.35.

We also separately constructed the universe for four structural classes to assess differences among their conformational spaces. For this study, we specifically examined the first 10 PC axes of each structural class because the 10 PC axes are more important than the other axes with respect to capturing the differences in the conformational axes. Although the eigenvectors from the same structural class are mutually uncorrelated (i.e., ***v***^*x*^_*i*_·***v***^*x*^_*j *_= 0, where *i *≠ *j *and *x *= α, β, α/β, or α+β), the eigenvectors from different structural classes might have some correlation (i.e., ***v***^*x*^_*i*_·***v***^*y*^_*j *_≠ 0, where *x *≠ *y*). The PC axis is defined as the conformational component specific to the structural class when a PC axis from a structural class has no similarity to the first 20 PC axes from the other structural classes with a correlation coefficient > 0.8 (i.e. ***v***^*x*^_*i*_·***v***^*y*^_*j *_> 0.8).

## Authors' contributions

This study was conceived and carried out by KI, who also analyzed the results and drafted the manuscript. HT approved the study and participated in the discussion. JH participated in the design and coordination of the study. He also helped to write the manuscript. KT participated in the design and discussions of the study and wrote the manuscript. KI and JH developed the methodology. All authors read and approved the final manuscript.

## Supplementary Material

Additional file 1 Origins of segments in the cluster of 30 residue length. Distributions of the origins of segments in the cluster of the universe of 30 residues length are shown. Click here for file

Additional file 2 Correlation with the first 10 PC axes of α/β class of the medium (26 residue) segments. Maximal correlation coefficients between the first 10 PC axes of α/β class and PC axes of the other three structural classes are shown. Click here for file

Additional file 3 Class-specific region for α/β segments on the *PC*^*α*/*β*^*8*-*PC*^*α*/*β*^*3 *plane. Distributions of segments of α/β structural class proteins for the medium length are shown. Click here for file

Additional file 4 Discrimination of segments from the α/β structural class in the *PC*^*α*/*β*^*8*-*PC*^*α*/*β*^*3 *plane. Specificity and coverage rates of segments of α/β structural class proteins in the *PC*^*α*/*β*^*8*-*PC*^*α*/*β*^*3 *plane are presented. Click here for file

Additional file 5 List of PDB ids used in this study. The PDB and SCOP IDs of proteins used in this study are listed. Click here for file
