# Supplementary material for: Protein-segment universe exhibiting transitions at intermediate segment length in conformational subspaces
Source: BMC Struct Biol. 2008 Aug 13;8:37. doi: 10.1186/1472-6807-8-37 (PMC2529298; doi:10.1186/1472-6807-8-37)
Supplement: Additional file 2 — Correlation with the first 10 PC axes of α/β class of the medium (26 residue) segments. Maximal correlation coefficients between the first 10 PC axes of α/β class and PC axes of the other three structural classes are shown. [file 1472-6807-8-37-S2.pdf]

Correlation with the first 10 PC axes of α/β class of the medium (26 residue) segments

The maximal correlation coefficient and PC axis (in parentheses) among the first 20 PC axes from the other three structural classes are shown with the first 10 PC axes of α/β class of the medium (26 residue) segments. Correlation coefficients greater than 0.8 are presented in bold typeface.

class	PC axes									
α/β	1st	2nd	3rd	4th	5th	6th	7th	8th	9th	10th
all-α	**0.972(1)**	**0.955(2)**	**0.952(3)**	**0.932(4)**	**0.803(6)**	0.771(5)	**0.884(7)**	0.665(11)	**0.831(9)**	0.762(8)
all-β	**0.984(1)**	**0.982(2)**	**0.969(3)**	**0.928(5)**	**0.920(4)**	**0.818(7)**	**0.943(6)**	0.725(9)	**0.821(8)**	0.617(11)
α+β	**0.997(1)**	**0.994(2)**	**0.960(3)**	**0.936(4)**	**0.973(5)**	**0.910(7)**	**0.904(6)**	0.732(10)	0.712(9)	0.670(10)
