# Supplementary material for: Protein-segment universe exhibiting transitions at intermediate segment length in conformational subspaces
Source: BMC Struct Biol. 2008 Aug 13;8:37. doi: 10.1186/1472-6807-8-37 (PMC2529298; doi:10.1186/1472-6807-8-37)
Supplement: Additional file 3 — Class-specific region for α/β segments on the PCα/β8-PCα/β3 plane. Distributions of segments of α/β structural class proteins for the medium length are shown. [file 1472-6807-8-37-S3.pdf]

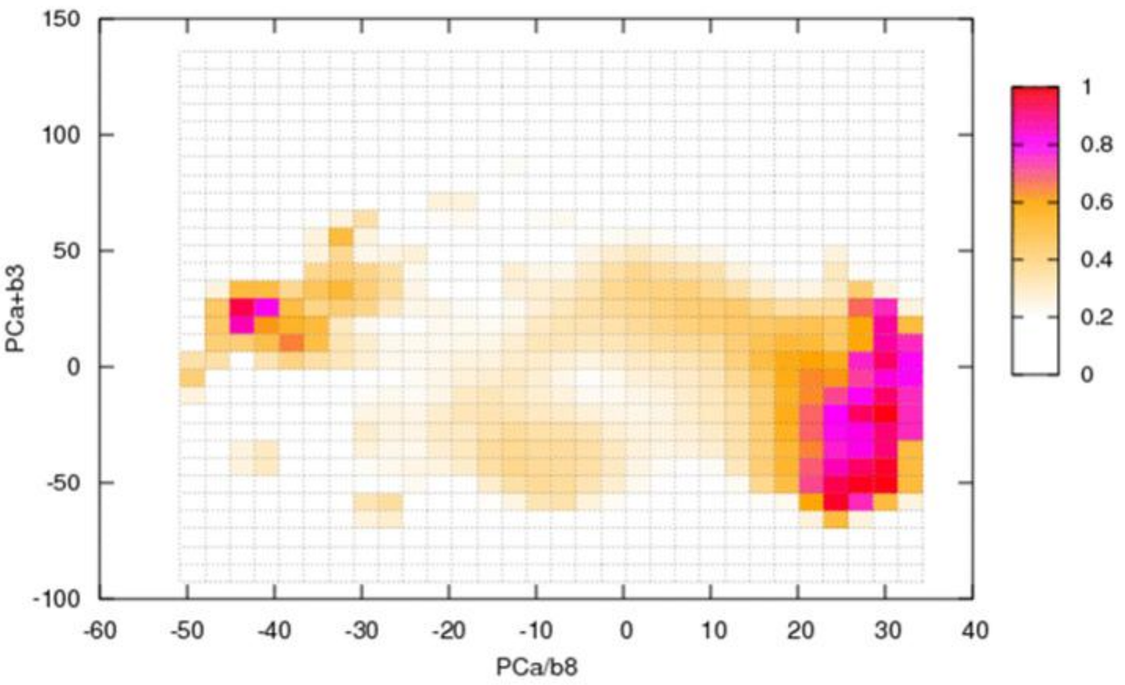

Class-specific region for α/β segments on the $PC^{\alpha/\beta}8$-$PC^{\alpha+\beta}3$ plane

The $PC^{\alpha/\beta}8$-$PC^{\alpha+\beta}3$ plane segregates α/β segments from the other class segments. The $PC^{\alpha/\beta}8$-$PC^{\alpha+\beta}3$ plane is divided into 30 × 30 bins. Color denotes the frequency of α/β segments in each bin, which is calculated as the number of segments derived from α/β proteins in a given bin divided by the number of all segments in the bin.
