# Supplementary material for: Protein-segment universe exhibiting transitions at intermediate segment length in conformational subspaces
Source: BMC Struct Biol. 2008 Aug 13;8:37. doi: 10.1186/1472-6807-8-37 (PMC2529298; doi:10.1186/1472-6807-8-37)
Supplement: Additional file 5 — List of PDB ids used in this study. The PDB and SCOP IDs of proteins used in this study are listed. [file 1472-6807-8-37-S5.pdf]

Table S1: List of PDB ids used in this study.

No	PDB id	Chan id	Scop Class	SCOP sccs number
1	1dlw	a	All alpha	a.1.1.1
2	2pgd		All alpha	a.100.1.1
3	1utg		All alpha	a.101.1.1
4	1gai		All alpha	a.102.1.1
5	1csh		All alpha	a.103.1.1
6	1dz4	a	All alpha	a.104.1.1
7	1dd3	a	All alpha	a.108.1.1
8	1iie	a	All alpha	a.109.1.1
9	2abd		All alpha	a.11.1.1
10	1aor	a	All alpha	a.110.1.1
11	1d2t	a	All alpha	a.111.1.1
12	1sig		All alpha	a.112.1.1
13	1ewq	a	All alpha	a.113.1.1
14	1f5n	a	All alpha	a.114.1.1
15	1bvp	1	All alpha	a.115.1.1
16	1tx4	a	All alpha	a.116.1.1
17	1bkd	s	All alpha	a.117.1.1
18	1qc7	a	All alpha	a.118.14.1
19	1yge		All alpha	a.119.1.1
20	1kdx	a	All alpha	a.12.1.1
21	1c1k	a	All alpha	a.120.1.1
22	2tct		All alpha	a.121.1.1
23	1fm9	a	All alpha	a.123.1.1
24	1ah7		All alpha	a.124.1.1
25	1f0j	a	All alpha	a.125.1.1
26	1bm0	a	All alpha	a.126.1.1
27	1jsw	a	All alpha	a.127.1.1
28	1uby		All alpha	a.128.1.1
29	1oel	a	All alpha	a.129.1.1
30	1lre		All alpha	a.13.1.1
31	1ecm	a	All alpha	a.130.1.1
32	1ppr	m	All alpha	a.131.1.1
33	1dvg	a	All alpha	a.132.1.1

34	1poc		All alpha	a.133.1.1
35	1bxm		All alpha	a.134.1.1
36	1g8q	a	All alpha	a.135.1.1
37	1dvo	a	All alpha	a.136.1.1
38	3cyr		All alpha	a.138.1.1
39	1daq	a	All alpha	a.139.1.1
40	1h9e	a	All alpha	a.140.1.1
41	1ijx	a	All alpha	a.141.1.1
42	1h99	a	All alpha	a.142.1.1
43	1i6v	e	All alpha	a.143.1.1
44	1g9l	a	All alpha	a.144.1.1
45	1g8e	a	All alpha	a.145.1.1
46	1h6o	a	All alpha	a.146.1.1
47	1k1f	a	All alpha	a.147.1.1
48	1k8k	e	All alpha	a.148.1.1
49	1i4s	a	All alpha	a.149.1.1
50	1tba	a	All alpha	a.15.1.1
51	1jr5	a	All alpha	a.150.1.1
52	1gu9	a	All alpha	a.152.1.1
53	1l8w	a	All alpha	a.154.1.1
54	1fjg	m	All alpha	a.156.1.1
55	1fs1	b	All alpha	a.157.1.1
56	1ail		All alpha	a.16.1.1
57	1f5a	a	All alpha	a.160.1.1
58	1luj	b	All alpha	a.161.1.1
59	1m6n	a	All alpha	a.162.1.1
60	1j0t	a	All alpha	a.163.1.1
61	1iyr	a	All alpha	a.164.1.1
62	1k5o	a	All alpha	a.165.1.1
63	1mlv	a	All alpha	a.166.1.1
64	1m5y	a	All alpha	a.167.1.1
65	1gzs	b	All alpha	a.168.1.1
66	1mi1	a	All alpha	a.169.1.1
67	2hp8		All alpha	a.17.1.1
68	1iyj	b	All alpha	a.170.1.1
69	1m6n	a	All alpha	a.172.1.1

70	1miv	a	All alpha	a.173.1.1
71	1k6k	a	All alpha	a.174.1.1
72	1m98	a	All alpha	a.175.1.1
73	1k87	a	All alpha	a.176.1.1
74	2end		All alpha	a.18.1.1
75	2lis	a	All alpha	a.19.1.1
76	1grj		All alpha	a.2.1.1
77	1lbu		All alpha	a.20.1.1
78	1ckt	a	All alpha	a.21.1.1
79	1hq3	a	All alpha	a.22.1.1
80	1fpo	a	All alpha	a.23.1.1
81	1bz4	a	All alpha	a.24.1.1
82	1dvb	a	All alpha	a.25.1.1
83	1rhg	a	All alpha	a.26.1.1
84	1a8h		All alpha	a.27.1.1
85	1acp		All alpha	a.28.1.1
86	1buc	a	All alpha	a.29.3.1
87	1c75	a	All alpha	a.3.1.1
88	1nkd		All alpha	a.30.1.1
89	1oct	c	All alpha	a.35.1.1
90	1skn	p	All alpha	a.37.1.1
91	1hlo	a	All alpha	a.38.1.1
92	4icb		All alpha	a.39.1.1
93	2hdd	a	All alpha	a.4.1.1
94	1bkr	a	All alpha	a.40.1.1
95	1a26		All alpha	a.41.1.1
96	1ycq	a	All alpha	a.42.1.1
97	1fvk	a	All alpha	a.44.1.1
98	1pgt	a	All alpha	a.45.1.1
99	1bmt	a	All alpha	a.46.1.1
100	1bf5	a	All alpha	a.47.1.1
101	2cbl	a	All alpha	a.48.1.1
102	1f6v	a	All alpha	a.49.1.1
103	1kjs		All alpha	a.50.1.1
104	2occ	h	All alpha	a.51.1.1
105	1hyp		All alpha	a.52.1.1

106	1adt		All alpha	a.54.1.1
107	1ihf	a	All alpha	a.55.1.1
108	1dgj	a	All alpha	a.56.1.1
109	1dj8	a	All alpha	a.57.1.1
110	1af7		All alpha	a.58.1.1
111	1e91	a	All alpha	a.59.1.1
112	1bqv		All alpha	a.60.1.1
113	1hiw	a	All alpha	a.61.1.1
114	1qgt	a	All alpha	a.62.1.1
115	1aep		All alpha	a.63.1.1
116	1nkl		All alpha	a.64.1.1
117	1ala		All alpha	a.65.1.1
118	1tad	a	All alpha	a.66.1.1
119	1ej5	a	All alpha	a.68.1.1
120	1e79	a	All alpha	a.69.1.1
121	2spc	a	All alpha	a.7.1.1
122	1abv		All alpha	a.70.1.1
123	1g7d	a	All alpha	a.71.1.1
124	1dvk	a	All alpha	a.72.1.1
125	1ak4	c	All alpha	a.73.1.1
126	1jsu	b	All alpha	a.74.1.1
127	1hus		All alpha	a.75.1.1
128	2dtr		All alpha	a.76.1.1
129	1ngr		All alpha	a.77.1.2
130	1hw1	a	All alpha	a.78.1.1
131	1eyv	a	All alpha	a.79.1.1
132	1dee	g	All alpha	a.8.1.1
133	1a5t		All alpha	a.80.1.1
134	1b79	a	All alpha	a.81.1.1
135	1crk	a	All alpha	a.83.1.1
136	1al0	1	All alpha	a.84.1.1
137	1lla		All alpha	a.86.1.1
138	1dbh	a	All alpha	a.87.1.1
139	1bou	a	All alpha	a.88.1.1
140	1mro	a	All alpha	a.89.1.1
141	1bgf		All alpha	a.90.1.1

142	1agr	e	All alpha	a.91.1.1
143	1a9x	a	All alpha	a.92.1.1
144	1llp		All alpha	a.93.1.1
145	1kqs	o	All alpha	a.94.1.1
146	1aa7	a	All alpha	a.95.1.1
147	2abk		All alpha	a.96.1.1
148	1gln		All alpha	a.97.1.1
149	1rlr		All alpha	a.98.1.1
150	1dnp	a	All alpha	a.99.1.1
151	1neu		All beta	b.1.1.1
152	2bpa	1	All beta	b.10.1.1
153	1ija	a	All beta	b.100.1.1
154	1e44	b	All beta	b.101.1.1
155	1fjr	a	All beta	b.102.1.1
156	1fc5	a	All beta	b.103.1.1
157	1hhn	a	All beta	b.104.1.1
158	1hd8	a	All beta	b.105.1.1
159	1k28	d	All beta	b.106.1.1
160	1ear	a	All beta	b.107.1.1
161	1h6w	.	All beta	b.108.1.1
162	1h8g	a	All beta	b.109.1.1
163	1amm		All beta	b.11.1.1
164	1jch	a	All beta	b.110.1.1
165	1k8h	a	All beta	b.111.1.1
166	1js8	a	All beta	b.112.1.1
167	1ee8	a	All beta	b.113.1.1
168	1m1g	a	All beta	b.114.1.1
169	1gzt	a	All beta	b.115.1.1
170	1mkf	a	All beta	b.116.1.1
171	1lnz	a	All beta	b.117.1.1
172	1o70	a	All beta	b.118.1.1
173	1ko6	.	All beta	b.119.1.1
174	1yge		All beta	b.12.1.1
175	1o75	a	All beta	b.120.1.1
176	1pgs		All beta	b.13.1.1
177	1df0	a	All beta	b.14.1.1

178	1shs	a	All beta	b.15.1.1
179	1slu	a	All beta	b.16.1.1
180	1beh	a	All beta	b.17.1.1
181	1gof		All beta	b.18.1.1
182	1bvp	1	All beta	b.19.1.1
183	1f0l	a	All beta	b.2.1.1
184	1aol		All beta	b.20.1.1
185	1knb		All beta	b.21.1.1
186	1aly		All beta	b.22.1.1
187	1sfp		All beta	b.23.1.1
188	1cb8	a	All beta	b.24.1.1
189	1aun		All beta	b.25.1.1
190	1ygs		All beta	b.26.1.1
191	1cq3	a	All beta	b.27.1.1
192	1p35	a	All beta	b.28.1.1
193	1nls		All beta	b.29.1.1
194	1cgt		All beta	b.3.1.1
195	1dp0	a	All beta	b.30.5.1
196	1es6	a	All beta	b.31.1.1
197	1qex	a	All beta	b.32.1.1
198	1rie		All beta	b.33.1.1
199	1aon	o	All beta	b.35.1.1
200	1pdr		All beta	b.36.1.1
201	1g3p		All beta	b.37.1.1
202	1b34	a	All beta	b.38.1.1
203	1whi		All beta	b.39.1.1
204	1c3g	a	All beta	b.4.1.1
205	1ey4	a	All beta	b.40.1.1
206	1dxr	h	All beta	b.41.1.1
207	1bfg		All beta	b.42.1.1
208	1fnd		All beta	b.43.4.2
209	1efc	a	All beta	b.44.1.1
210	1flm	a	All beta	b.45.1.1
211	1fmt	a	All beta	b.46.1.1
212	1arb		All beta	b.47.1.1
213	1bco		All beta	b.48.1.1

214	1e79	a	All beta	b.49.1.1
215	1hoe		All beta	b.5.1.1
216	1daz	c	All beta	b.50.1.1
217	1ile		All beta	b.51.1.1
218	2eng		All beta	b.52.1.1
219	1dfu	p	All beta	b.53.1.1
220	1cl3	a	All beta	b.54.1.1
221	1mai		All beta	b.55.1.1
222	1wpo	a	All beta	b.57.1.1
223	1a49	a	All beta	b.58.1.1
224	1fu1	a	All beta	b.59.1.1
225	1aac		All beta	b.6.1.1
226	1hbq		All beta	b.60.1.1
227	1swu	a	All beta	b.61.1.1
228	2cpl		All beta	b.62.1.1
229	1jsg		All beta	b.63.1.1
230	1c39	a	All beta	b.64.1.1
231	1f3u	a	All beta	b.65.1.1
232	1hxn		All beta	b.66.1.1
233	1tl2	a	All beta	b.67.1.1
234	1dil		All beta	b.68.1.1
235	1gof		All beta	b.69.1.1
236	1qas	a	All beta	b.7.1.1
237	4aah	a	All beta	b.70.1.1
238	1vjs		All beta	b.71.1.1
239	1dkg	a	All beta	b.73.1.1
240	1hcb		All beta	b.74.1.1
241	4bcl		All beta	b.75.1.1
242	1osp	o	All beta	b.76.1.1
243	1vmo	a	All beta	b.77.1.1
244	1jpc		All beta	b.78.1.1
245	1kap	p	All beta	b.79.1.1
246	1czy	a	All beta	b.8.1.1
247	1air		All beta	b.80.1.1
248	1lxa		All beta	b.81.1.1
249	1dzr	a	All beta	b.82.1.1

250	1qiu	a	All beta	b.83.1.1
251	1bdo		All beta	b.84.1.1
252	1hg7	a	All beta	b.85.1.1
253	1at0		All beta	b.86.1.1
254	1umu	a	All beta	b.87.1.1
255	1hxr	a	All beta	b.88.1.1
256	3ezm	a	All beta	b.89.1.1
257	2bn2	a	All beta	b.9.1.1
258	1lkt	a	All beta	b.90.1.1
259	1qqh	a	All beta	b.91.1.1
260	1fwf	c	All beta	b.92.1.1
261	1aqt		All beta	b.93.1.1
262	1f35	a	All beta	b.94.1.1
263	1g13	a	All beta	b.95.1.1
264	1i9b	a	All beta	b.96.1.1
265	1iaz	a	All beta	b.97.1.1
266	1hs6	a	All beta	b.98.1.1
267	1tph	1	Alpha/beta	c.1.1.1
268	2bnh		Alpha/beta	c.10.1.1
269	1ig0	a	Alpha/beta	c.100.1.1
270	1f75	a	Alpha/beta	c.101.1.1
271	1ihn	a	Alpha/beta	c.103.1.1
272	1jzt	a	Alpha/beta	c.104.1.1
273	1ejj	a	Alpha/beta	c.105.1.1
274	1j9j	a	Alpha/beta	c.106.1.1
275	1i74	a	Alpha/beta	c.107.1.1
276	1zrn		Alpha/beta	c.108.1.1
277	1aq2		Alpha/beta	c.109.1.1
278	1ds9	a	Alpha/beta	c.11.1.1
279	1jke	a	Alpha/beta	c.110.1.1
280	1jw9	b	Alpha/beta	c.111.1.1
281	1k30	a	Alpha/beta	c.112.1.1
282	1jr2	a	Alpha/beta	c.113.1.1
283	1l1s	a	Alpha/beta	c.114.1.1
284	1kjn	a	Alpha/beta	c.115.1.1
285	1ipa	a	Alpha/beta	c.116.1.1

286	1gr8	a	Alpha/beta	c.117.1.1
287	1o0u	a	Alpha/beta	c.118.1.1
288	1mgp	a	Alpha/beta	c.119.1.1
289	1kd1	p	Alpha/beta	c.12.1.1
290	1aua		Alpha/beta	c.13.1.1
291	1tyf	a	Alpha/beta	c.14.1.1
292	1cdz	a	Alpha/beta	c.15.1.1
293	1rvv	a	Alpha/beta	c.16.1.1
294	1cp3	a	Alpha/beta	c.17.1.1
295	1akz		Alpha/beta	c.18.1.1
296	1mla		Alpha/beta	c.19.1.1
297	1ee2	a	Alpha/beta	c.2.1.1
298	1g7s	a	Alpha/beta	c.20.1.1
299	1k8a	k	Alpha/beta	c.21.1.1
300	1dmg	a	Alpha/beta	c.22.1.1
301	3chy		Alpha/beta	c.23.1.1
302	1a9x	a	Alpha/beta	c.24.1.1
303	1fnd		Alpha/beta	c.25.1.1
304	2ts1		Alpha/beta	c.26.1.1
305	2tpt		Alpha/beta	c.27.1.1
306	1dnp	a	Alpha/beta	c.28.1.1
307	1djn	a	Alpha/beta	c.3.1.1
308	1dv1	a	Alpha/beta	c.30.1.1
309	1dhs		Alpha/beta	c.31.1.1
310	1fsz		Alpha/beta	c.32.1.1
311	1nba	a	Alpha/beta	c.33.1.1
312	1e20	a	Alpha/beta	c.34.1.1
313	1dea	a	Alpha/beta	c.35.1.1
314	1pyd	a	Alpha/beta	c.36.1.1
315	1gky		Alpha/beta	c.37.1.1
316	1ble		Alpha/beta	c.38.1.1
317	1d0v	a	Alpha/beta	c.39.1.1
318	1djn	a	Alpha/beta	c.4.1.1
319	1chd		Alpha/beta	c.40.1.1
320	1cse	e	Alpha/beta	c.41.1.1
321	1d3v	a	Alpha/beta	c.42.1.1

322	3cla		Alpha/beta	c.43.1.1
323	1phr		Alpha/beta	c.44.1.1
324	1vhr	a	Alpha/beta	c.45.1.1
325	1c25		Alpha/beta	c.46.1.1
326	2trx	a	Alpha/beta	c.47.1.1
327	1trk	a	Alpha/beta	c.48.1.1
328	1pkm		Alpha/beta	c.49.1.1
329	2uag	a	Alpha/beta	c.5.1.1
330	1lam		Alpha/beta	c.50.1.1
331	1kmm	a	Alpha/beta	c.51.1.1
332	1ckq	a	Alpha/beta	c.52.1.1
333	1gdt	a	Alpha/beta	c.53.1.1
334	1pdo		Alpha/beta	c.54.1.1
335	1bup	a	Alpha/beta	c.55.1.1
336	1cfz	a	Alpha/beta	c.56.1.1
337	1di6	a	Alpha/beta	c.57.1.1
338	1bgv	a	Alpha/beta	c.58.1.1
339	2uag	a	Alpha/beta	c.59.1.1
340	1tml		Alpha/beta	c.6.1.1
341	1qhf	a	Alpha/beta	c.60.1.1
342	1nul	a	Alpha/beta	c.61.1.1
343	1lfa	a	Alpha/beta	c.62.1.1
344	1poi	a	Alpha/beta	c.63.1.2
345	1b0p	a	Alpha/beta	c.64.1.1
346	2gar		Alpha/beta	c.65.1.1
347	1vid		Alpha/beta	c.66.1.1
348	7aat	a	Alpha/beta	c.67.1.1
349	1qg8	a	Alpha/beta	c.68.1.1
350	2ack		Alpha/beta	c.69.1.1
351	1cm5	a	Alpha/beta	c.7.1.1
352	2mas	a	Alpha/beta	c.70.1.1
353	1ra9		Alpha/beta	c.71.1.1
354	1rkd		Alpha/beta	c.72.1.1
355	1b7b	a	Alpha/beta	c.73.1.1
356	1e4c	p	Alpha/beta	c.74.1.1
357	1ed8	a	Alpha/beta	c.76.1.1

358	1xaa		Alpha/beta	c.77.1.1
359	1ekx	a	Alpha/beta	c.78.1.1
360	1ttq	b	Alpha/beta	c.79.1.1
361	1dik		Alpha/beta	c.8.1.1
362	1moq		Alpha/beta	c.80.1.1
363	1eu1	a	Alpha/beta	c.81.1.1
364	1ad3	a	Alpha/beta	c.82.1.1
365	7acn		Alpha/beta	c.83.1.1
366	3pmg	a	Alpha/beta	c.84.1.1
367	1fui	a	Alpha/beta	c.85.1.1
368	1qpg		Alpha/beta	c.86.1.1
369	1c3j	a	Alpha/beta	c.87.1.1
370	4eca	a	Alpha/beta	c.88.1.1
371	1pfk	a	Alpha/beta	c.89.1.1
372	1cbf		Alpha/beta	c.90.1.1
373	1jb1	a	Alpha/beta	c.91.1.2
374	1doz	a	Alpha/beta	c.92.1.1
375	2dri		Alpha/beta	c.93.1.1
376	1jet	a	Alpha/beta	c.94.1.1
377	1afw	a	Alpha/beta	c.95.1.1
378	1feh	a	Alpha/beta	c.96.1.1
379	1aln		Alpha/beta	c.97.1.1
380	1gg4	a	Alpha/beta	c.98.1.1
381	1hi9	a	Alpha/beta	c.99.1.1
382	1rge	a	Alpha+beta	d.1.1.2
383	1bb8		Alpha+beta	d.10.1.1
384	1div		Alpha+beta	d.100.1.1
385	1e3h	a	Alpha+beta	d.101.1.1
386	1efn	b	Alpha+beta	d.102.1.1
387	1cby		Alpha+beta	d.103.1.1
388	1sry	a	Alpha+beta	d.104.1.1
389	1qts	a	Alpha+beta	d.105.1.1
390	1qnd	a	Alpha+beta	d.106.1.1
391	1eq6	a	Alpha+beta	d.107.1.1
392	1b87	a	Alpha+beta	d.108.1.1
393	2vik		Alpha+beta	d.109.1.1

394	1qme	a	Alpha+beta	d.11.1.1
395	1pne		Alpha+beta	d.110.1.1
396	1cfe		Alpha+beta	d.111.1.1
397	1a6j	a	Alpha+beta	d.112.1.1
398	1mut		Alpha+beta	d.113.1.1
399	1ush		Alpha+beta	d.114.1.1
400	1hru	a	Alpha+beta	d.115.1.1
401	1dbx	a	Alpha+beta	d.116.1.1
402	1qqq	a	Alpha+beta	d.117.1.1
403	1lba		Alpha+beta	d.118.1.1
404	1m90	t	Alpha+beta	d.12.1.1
405	1cyo		Alpha+beta	d.120.1.1
406	1vcc		Alpha+beta	d.121.1.1
407	1amw		Alpha+beta	d.122.1.1
408	1ixm	a	Alpha+beta	d.123.1.1
409	1bol	a	Alpha+beta	d.124.1.1
410	1c4k	a	Alpha+beta	d.125.1.1
411	1g61	a	Alpha+beta	d.126.1.1
412	1chm	a	Alpha+beta	d.127.1.1
413	1lgr		Alpha+beta	d.128.1.1
414	1cdw	a	Alpha+beta	d.129.1.1
415	4rhn		Alpha+beta	d.13.1.1
416	1mxa		Alpha+beta	d.130.1.1
417	2pol	a	Alpha+beta	d.131.1.1
418	1dgj	a	Alpha+beta	d.133.1.1
419	1aop		Alpha+beta	d.134.1.1
420	1duj	a	Alpha+beta	d.135.1.1
421	1byr	a	Alpha+beta	d.136.1.1
422	1ckv		Alpha+beta	d.137.1.1
423	1pys	b	Alpha+beta	d.138.1.1
424	1cli	a	Alpha+beta	d.139.1.1
425	1fnm	a	Alpha+beta	d.14.1.1
426	1sei	a	Alpha+beta	d.140.1.1
427	1rl6	a	Alpha+beta	d.141.1.1
428	1gsa		Alpha+beta	d.142.1.1
429	1a48		Alpha+beta	d.143.1.1

430	1hcl		Alpha+beta	d.144.1.1
431	1e8g	a	Alpha+beta	d.145.1.1
432	1uxy		Alpha+beta	d.146.1.1
433	1qlm	a	Alpha+beta	d.147.1.1
434	1c4z	a	Alpha+beta	d.148.1.1
435	2ahj	a	Alpha+beta	d.149.1.1
436	1ubi		Alpha+beta	d.15.1.1
437	1qr0	a	Alpha+beta	d.150.1.1
438	1ako		Alpha+beta	d.151.1.1
439	1aor	a	Alpha+beta	d.152.1.1
440	1gdo	a	Alpha+beta	d.153.1.1
441	1b65	a	Alpha+beta	d.154.1.1
442	1pya	.	Alpha+beta	d.155.1.1
443	1jen	.	Alpha+beta	d.156.1.1
444	2bc2	a	Alpha+beta	d.157.1.1
445	4kbp	a	Alpha+beta	d.159.1.1
446	3cox		Alpha+beta	d.16.1.1
447	1ems	a	Alpha+beta	d.160.1.1
448	1qdl	a	Alpha+beta	d.161.1.1
449	1mld	a	Alpha+beta	d.162.1.1
450	1aih	a	Alpha+beta	d.163.1.1
451	1mhd	a	Alpha+beta	d.164.1.1
452	1mrj		Alpha+beta	d.165.1.1
453	1lts	.	Alpha+beta	d.166.1.1
454	1bsz	a	Alpha+beta	d.167.1.1
455	1chu	a	Alpha+beta	d.168.1.1
456	1qdd	a	Alpha+beta	d.169.1.1
457	1mol	a	Alpha+beta	d.17.1.1
458	1by2		Alpha+beta	d.170.1.1
459	1fid		Alpha+beta	d.171.1.1
460	1g9m	g	Alpha+beta	d.172.1.1
461	1msk		Alpha+beta	d.173.1.1
462	1nos		Alpha+beta	d.174.1.1
463	1sox	a	Alpha+beta	d.176.1.1
464	1hyo	a	Alpha+beta	d.177.1.1
465	1toh		Alpha+beta	d.178.1.1

466	1dqa	a	Alpha+beta	d.179.1.1
467	1pcf	a	Alpha+beta	d.18.1.1
468	16vp	a	Alpha+beta	d.180.1.1
469	1bdf	a	Alpha+beta	d.181.1.1
470	1el6	a	Alpha+beta	d.182.1.1
471	1fh6	a	Alpha+beta	d.183.1.1
472	1qgw	a	Alpha+beta	d.184.1.1
473	1bcc	a	Alpha+beta	d.185.1.1
474	1hyw	a	Alpha+beta	d.186.1.1
475	1jb0	d	Alpha+beta	d.187.1.1
476	1gd8	a	Alpha+beta	d.188.1.1
477	1gd5	a	Alpha+beta	d.189.1.1
478	3fru	a	Alpha+beta	d.19.1.1
479	1fw9	a	Alpha+beta	d.190.1.1
480	1g2r	a	Alpha+beta	d.192.1.1
481	1hw7	a	Alpha+beta	d.193.1.1
482	1hq0	a	Alpha+beta	d.194.1.1
483	1huf	a	Alpha+beta	d.195.1.1
484	1fn9	a	Alpha+beta	d.196.1.1
485	1dl5	a	Alpha+beta	d.197.1.1
486	1jya	a	Alpha+beta	d.198.1.1
487	1kaf	a	Alpha+beta	d.199.1.1
488	1cns	a	Alpha+beta	d.2.1.1
489	2aak		Alpha+beta	d.20.1.1
490	1jv2	b	Alpha+beta	d.200.1.1
491	1jid	a	Alpha+beta	d.201.1.1
492	1hh2	p	Alpha+beta	d.202.1.1
493	1ji8	a	Alpha+beta	d.203.1.1
494	1imu	a	Alpha+beta	d.204.1.1
495	1is7	k	Alpha+beta	d.205.1.1
496	1jrm	a	Alpha+beta	d.206.1.1
497	1kq4	a	Alpha+beta	d.207.1.1
498	1jw3	a	Alpha+beta	d.208.1.1
499	1jbi	a	Alpha+beta	d.209.1.1
500	1bwz	a	Alpha+beta	d.21.1.1
501	1k92	a	Alpha+beta	d.210.1.1

502	1ycs	b	Alpha+beta	d.211.1.1
503	1tol	a	Alpha+beta	d.212.1.1
504	1lg7	a	Alpha+beta	d.213.1.1
505	1iqo	a	Alpha+beta	d.214.1.1
506	1gxj	a	Alpha+beta	d.215.1.1
507	1l9v	a	Alpha+beta	d.216.1.1
508	1h5p	a	Alpha+beta	d.217.1.1
509	1jaj	a	Alpha+beta	d.218.1.2
510	1a6q		Alpha+beta	d.219.1.1
511	1ema		Alpha+beta	d.22.1.1
512	1eul	a	Alpha+beta	d.220.1.1
513	1j57	a	Alpha+beta	d.221.1.1
514	1j8b	a	Alpha+beta	d.222.1.1
515	1mby	a	Alpha+beta	d.223.1.1
516	1mzg	a	Alpha+beta	d.224.1.1
517	1iwg	a	Alpha+beta	d.225.1.1
518	1ln0	a	Alpha+beta	d.226.1.1
519	1n2f	a	Alpha+beta	d.227.1.1
520	1lrr	a	Alpha+beta	d.228.1.1
521	1ni5	a	Alpha+beta	d.229.1.1
522	1c8z	a	Alpha+beta	d.23.1.1
523	2pil		Alpha+beta	d.24.1.1
524	1p32	a	Alpha+beta	d.25.1.1
525	1bkf		Alpha+beta	d.26.1.1
526	1fjg	p	Alpha+beta	d.27.1.1
527	1fjg	s	Alpha+beta	d.28.1.1
528	1kqs	w	Alpha+beta	d.29.1.1
529	2act		Alpha+beta	d.3.1.1
530	1b33	n	Alpha+beta	d.30.1.1
531	1qcs	a	Alpha+beta	d.31.1.1
532	1qip	a	Alpha+beta	d.32.1.1
533	1fx3	a	Alpha+beta	d.33.1.1
534	1bm8		Alpha+beta	d.34.1.1
535	1dk0	a	Alpha+beta	d.35.1.1
536	1eyq	a	Alpha+beta	d.36.1.1
537	1bvq	a	Alpha+beta	d.38.1.1

538	1cmi	a	Alpha+beta	d.39.1.1
539	7cei	b	Alpha+beta	d.4.1.1
540	1cse	i	Alpha+beta	d.40.1.1
541	1dgj	a	Alpha+beta	d.41.1.1
542	1buo	a	Alpha+beta	d.42.1.1
543	1efu	b	Alpha+beta	d.43.1.1
544	1i0h	a	Alpha+beta	d.44.1.1
545	1ctf		Alpha+beta	d.45.1.1
546	1ekt	a	Alpha+beta	d.46.1.1
547	1mms	a	Alpha+beta	d.47.1.1
548	2reb		Alpha+beta	d.48.1.1
549	1e8o	a	Alpha+beta	d.49.1.1
550	1dy5	a	Alpha+beta	d.5.1.1
551	1di2	a	Alpha+beta	d.50.1.1
552	1dt4	a	Alpha+beta	d.51.1.1
553	1fjg	c	Alpha+beta	d.53.1.1
554	1one	a	Alpha+beta	d.54.1.1
555	1bxe	a	Alpha+beta	d.55.1.1
556	1ghh	a	Alpha+beta	d.57.1.1
557	1fxd		Alpha+beta	d.58.1.1
558	1bxy	a	Alpha+beta	d.59.1.1
559	1ag2		Alpha+beta	d.6.1.1
560	1bow	a	Alpha+beta	d.60.1.1
561	1fsi	a	Alpha+beta	d.61.1.1
562	1f32	a	Alpha+beta	d.62.1.1
563	1d8i	a	Alpha+beta	d.63.1.1
564	2if1		Alpha+beta	d.64.1.1
565	1lbu		Alpha+beta	d.65.1.1
566	1fjg	d	Alpha+beta	d.66.1.2
567	1qf6	a	Alpha+beta	d.67.1.1
568	1tig		Alpha+beta	d.68.1.1
569	1kpt	a	Alpha+beta	d.70.1.1
570	1ev0	a	Alpha+beta	d.71.1.1
571	1dw9	a	Alpha+beta	d.72.1.1
572	3rub	s	Alpha+beta	d.73.1.1
573	1dcp	a	Alpha+beta	d.74.1.1

574	1a79	a	Alpha+beta	d.75.1.1
575	1gyf	a	Alpha+beta	d.76.1.1
576	1k9m	f	Alpha+beta	d.77.1.1
577	1eik	a	Alpha+beta	d.78.1.1
578	1qu9	a	Alpha+beta	d.79.1.1
579	1ejr	a	Alpha+beta	d.8.1.1
580	1otf	a	Alpha+beta	d.80.1.1
581	1gad	o	Alpha+beta	d.81.1.1
582	1oac	a	Alpha+beta	d.82.1.1
583	1ewf	a	Alpha+beta	d.83.1.1
584	3sic	i	Alpha+beta	d.84.1.1
585	1e6t	a	Alpha+beta	d.85.1.1
586	1ej1	a	Alpha+beta	d.86.1.1
587	3grs		Alpha+beta	d.87.1.1
588	1srs	a	Alpha+beta	d.88.1.1
589	1tbd		Alpha+beta	d.89.1.1
590	3il8		Alpha+beta	d.9.1.1
591	1nox		Alpha+beta	d.90.1.1
592	1dt9	a	Alpha+beta	d.91.1.1
593	1kuh		Alpha+beta	d.92.1.1
594	1lkk	a	Alpha+beta	d.93.1.1
595	1sph	a	Alpha+beta	d.94.1.1
596	1iba		Alpha+beta	d.95.1.1
597	1a8r	a	Alpha+beta	d.96.1.1
598	1puc		Alpha+beta	d.97.1.1
599	1jtg	b	Alpha+beta	d.98.1.1
600	1div		Alpha+beta	d.99.1.1
